# Vitiligo onset following anti-inflammatory biological therapy: a scoping review

**DOI:** 10.3389/fimmu.2026.1879414

**Published:** 2026-10-06

**Authors:** Maurizio Romagnuolo, Chiara Moltrasio, Luisa Carlotta Rossi, Gianluca Tavoletti, Angelo Valerio Marzano, Silvia Alberti Violetti

**Affiliations:** 1 Dermatology Unit, Fondazione IRCCS Ca’ Granda Ospedale Maggiore Policlinico, Milan, Italy; 2 Department of Medical Biotechnologies, University of Siena, Siena, Italy; 3 Department of Pathophysiology and Transplantation, Università degli Studi di Milano, Milan, Italy

**Keywords:** adalimumab, biological therapy, drug event, adverse, dupilumab, secukinumab, tumor necrosis factor inhibitors, vitiligo

## Abstract

**Introduction:**

Vitiligo is a chronic autoimmune depigmentation skin disorder characterized by the acquired loss of melanocytes, with a complex multifactorial pathophysiology. Among the potential predisposing factors, some drugs, including monoclonal antibodies, have been associated to disease onset.

**Methods:**

The aim of the present review is to summarize and discuss current literature findings in de novo vitiligo following biological targeting inflammatory cytokines.

**Results:**

Overall, 18 articles were considered eligible for the present review, and details of demographics, clinical characteristics, vitiligo type and localization, biological drug administered and treatment outcomes were reported.The mean age of the 39 patients was 43 years (±16.3), with a male predominance (62%). Tumor necrosis factor-alpha (TNFα) inhibitors (infliximab, adalimumab, certolizumab pegol) were the most frequent suspected drugs (>50% of patients), followed by anti-IL-17A (ixekizumab, secukinumab) and anti-IL-12/23 (ustekinumab). Four cases were linked to anti-IL-4/13 (dupilumab). The median onset of vitiligo was 5 months post-initiation (range 1.5–24), with one-third of cases involving the face and neck, and two cases of segmental vitiligo. Biologic discontinuation or switching occurred in 26% of cases. Regarding outcomes, 41% experienced stabilization, 36% achieved varying degrees of repigmentation, and 8% progressed.

**Discussion:**

While de novo vitiligo is reported as a potential adverse event of biological therapies, evidence for a direct causal relationship remains weak, with cases involving non-anti-TNFα agents appearing anecdotal. The co-occurrence of underlying autoimmune comorbidities in these patients points towards a coincidental rather than a causal relationship, highlighting the need for further research to fully elucidate this association.

## Introduction

Vitiligo is a chronic autoimmune depigmentation skin disorder hallmarked by the acquired loss of functional epidermal melanocytes, representing one of the most common dermatological diseases with an estimated global lifetime prevalence of 0.36% affected individuals (1). While vitiligo clinical presentation is usually straightforward, presenting with milky-white, non-scaling patches in the affected areas, its pathophysiology remains largely elusive (1). The current pathogenetic model is centered on the genetically predisposed vulnerability of melanocytes towards cellular oxidative stress, which is accompanied by local innate immunity activation, subsequently followed by an autoreactive adaptive immunity response with destruction of functional melanocytes (1, 2). Genetic predisposition represents a major risk factor for vitiligo onset, as attested by the family clustering of vitiligo cases and the more than 50 genetic susceptibility loci identified in genome-wide association studies (GWASs). These loci are mainly involved in melanocyte biology, oxidative stress pathways, and immune responses, with immune responses shared with other autoimmune diseases (1, 3). However, genetic twin studies showing a 23% concordance rate in vitiligo indicate that environmental factors could also play a role in disease onset (1, 4). Among the potential triggering factors, physical traumatism (Koebner phenomenon), exposure to phenolic compounds, and the patient’s reported psychological stress have all been linked to vitiligo onset (1). Regarding possible pharmacological triggering agents, the notion of systemic drug-induced or drug-associated vitiligo is still under debate, although strong evidence of vitiligo-like depigmentation following immune checkpoint inhibitors (ICIs) [such as programmed cell death 1 (PD1) and PD1-ligand (PD1/L) inhibitors (e.g., nivolumab, pembrolizumab, and atezolizumab)] and cyclin-dependent kinase 4/6 inhibitors (CDKis), such as palbociclib and ribociclib, are widely available (5–8). While the mechanism of action of ICIs is known to favor autoimmune-like diseases (including vitiligo), the pathophysiological mechanism underlying CDKi-associated vitiligo-like depigmentation is not yet elucidated, although vitiligo-like depigmentation is reported as a relatively common side effect and is also listed in the ribociclib summary of product characteristics (9, 10).

Conversely, literature data regarding *de novo* vitiligo associated with monoclonal antibodies targeting inflammatory cytokines are scarce and controversial, although in recent years, some reports have described vitiligo onset or progression during targeted therapies prescribed for inflammatory conditions (11).

The aim of the present review is to summarize and discuss current literature findings in *de novo* vitiligo following biological targeted therapies directed at inflammatory cytokines. The systematic search was conducted in the MEDLINE (via PubMed) and Web of Science (WOS) databases from October 2025 to 31 December 2025 using a combined strategy of free-text keywords and pharmacological descriptors with the following string: (“vitiligo”) AND (“biolog*” OR “mab*” OR “cept*” OR “anti-TNF”) AND (“induced” OR “paradoxical” OR “onset” OR “associat*”). Additionally, relevant keyword combinations were utilized, and the bibliography of selected articles was reviewed.

Title and abstract screening and subsequent full-text eligibility assessment were performed independently by two investigators (M.R. and G.T.) to minimize selection bias. Any discrepancies regarding study inclusion were resolved through consensus with a third investigator (C.M.). Only articles in English were considered eligible for the review. Cases in which vitiligo appeared in areas of a cleared skin disease (e.g., psoriasis and eczema), single case reports without clinical photos, and cases reporting a worsening of pre-existing vitiligo were excluded from the review. The article selection process is visually summarized in a Preferred Reporting System for Systematic reviews and Meta-Analyses (PRISMA)-compliant flow diagram (**Figure 1**).

**Figure 1:**
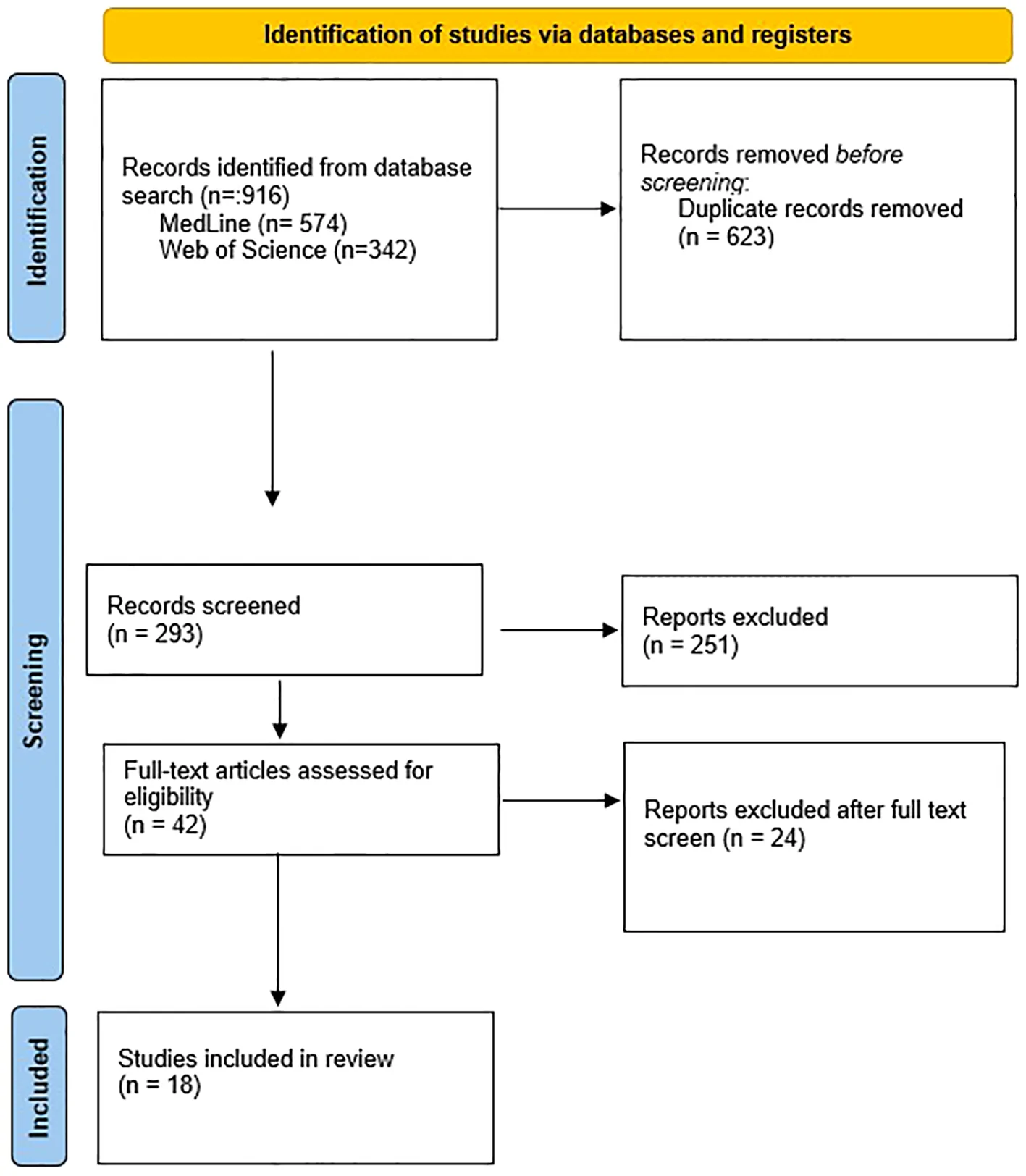
Flow diagram showing the study selection process (adapted from Page et al., 2021 available at prisma-statement.org).

## Results

Overall, 18 articles were considered for the review (12–29). General demographics, clinical characteristics, vitiligo type and localization, biological drug administered, and treatment outcomes are reported in **Table 1**, with median age and some of the key clinical findings summarized in **Table 2**.

**Table 1:** Demographic and clinical information regarding *de novo* vitiligo following biological therapies.

Author, year, reference	Sex, age	Drug	Indication	Potential vitiligo triggers	Vitiligo onset (months)	Vitiligo location	Treatment	Outcome (follow-up)
Ismail W.A. et al. (2011) (12)	M, 51	IFX	UC	No	1.5	Face	- Infliximab discontinuation - Tacrolimus ointment - Excimer laser	CR (1 month)
Posada C. et al. (2011) (13)	F, 54	ADA	CD	No	8	Trunk and upper extremities	n/r	n/r
Carvalho et al. (2014) (14)	F, 46	IFX	RA	Diabetes; ANA 1:160 mixed pattern	8	Segmental, trunk, and left upper limb	- 8-Methoxypsoralen solution 0.0001% - Dexamethasone cream	Stability (n/r)
Jung J.M. et al. (2015) (15)	F, 39	ADA	CD	No	12	Hands, feet, and limbs	- Topical tacrolimus - Excimer laser	Stability (n/r)
Luber et al. (2017) (16)	M, 83	IFX	CD	n/r	6	Trunk and limbs	- Tacrolimus ointment 0.1% Calcipotriol/betamethasone ointment twice daily	Moderate improvement (n/r)
Ryu et al. (2017) (17)	M, 34	IFX	UC	No	4	Right mandibular area (segmental)	- Excimer laser	Stability (n/r)
Mèry-Bossard et al. (2017) (18)	M/F (n = 11/7), 42.8 ± 12.8	ADA (n = 8) IFX (n = 3) CZP (n = 3) UST (n = 3) Abatacept (n = 1) SEC (n = 1)	PSO (n = 8) RA (n = 4) AS (n = 4) UC (n = 1) Uveitis (n = 1)	Diabetes (n = 2)	13.9 ± 16.5 (median 10)	Trunk and limbs (n = 10) Face/head (n = 3) Poliosis (n = 1) Leucotrichia (n = 1)	- Infliximab discontinuation, n = 2 - Abatacept discontinuation, n = 1 - Switching adalimumab to etanercept, n = 2 - Switching infliximab to ustekinumab, n = 1	Stability (n = 9; 52.9%) Repigmentation (n = 6; 35.2%) Progression (n = 2; 11.8%)
Pirro et al. (2019) (19)	M. 48	IXE	PSO	No	3	Legs, feet, and hands	- Tacrolimus ointment	50% improvement (4 months)
Gedikli et al. (2020) (20)	M, 33	UST	PsA	No	3.5	Fingers	- Switch to adalimumab	Stability (6 months)
Phan et al. (2020) (21)	F, 24	ADA	HS	no	3	Posterior legs and back	n/r	n/r
Nieto-Benito et al. (2020) (22)	M, 39	SEC	PSO	No	24	Axillae, neck, arms, and feet; halo nevus	- Tacrolimus ointment	Stability (18 months)
	F, 43	SEC	PSO	No	12	Axillae	- Tacrolimus ointment	Stability (16 months)
Marasca et al. (2021) (23)	M, 53	IXE	PSO	No	3	Face	- Topical calcineurin inhibitor	n/r
Yang H.J. et al. (2021) (24)	M, 22	ADA	Healthy volunteer	No	1.5	Neck, chest	- Excimer laser	Improvement (5 months)
Eker et al. (2021) (25)	M, 71	IXE	PSO	No	3	Face	- Topical steroid; topical pimecrolimus	n/r
Pathmarajah et al. (2022) (26)	M, 36	IXE	PSO	No	11	Trunk, limbs, face	n/r	n/r
Su H.J. et al. (2022) (27)	F, 72	IXE	PSO	No	4	Face	- Ixekizumab discontinuation - Excimer laser - Cyclosporine	75% repigmentation (3 months)
Ren et al. (2023) (28)	M/F (2/2)	DUPI	AD	n/r	4 (median)	- Cheeks and neck - Hairline, neck, and chest - Hands and feet - Hands, legs, and feet	- Dupilumab discontinuation, n = 2 - Tacrolimus 0.1%, n = 1 - Mometasone 0.1% ointment and Narrow band- Ultraviolet B (Nb-UVD), n = 1 - Triamcinolone acetonide 0.1% and tacrolimus ointment 0.1%, n = 1	Repigmentation (n = 3, 75%) Progression (n = 1, 25%)
Değirmenci et al. (2024) (29)	M, 34	ADA	Uveitis	No	5	Left mandibular area	- Topical tacrolimus	Stability (3 months)

M, male; IFX, infliximab; UC, ulcerative colitis; CR, complete remission; F, female; CD, Crohn’s disease; n/r, not reported; RA, rheumatoid arthritis; ADA, adalimumab; n, number of patients; CZP, certolizumab pegol; UST, ustekinumab; SEC, secukinumab; AS, ankylosing spondylitis; PSO, psoriasis; PsA, psoriatic arthritis; IXE, ixekizumab; HS, hidradenitis suppurativa; DUPI, dupilumab; AD, atopic dermatitis.

**Table 2:** Summary of the main findings of the included cases.

Number of patients	39
Sex	M = 24 (62%) F = 15 (38%)
Age	43 ± 16.3 M = 39 (range, 22–83) F = 44.5 (range, 24–72)
Biological drug	Infliximab = 6 (16%) Adalimumab = 13 (33%) Certolizumab pegol = 3 (8%) Ixekizumab = 5 (13%) Ustekinumab = 4 (10%) Secukinumab = 3 (8%) Dupilumab = 4 (10%) Abatacept = 1 (2%)
Vitiligo onset (months, median)	5 (range, 1.5–24)
Vitiligo location	Face and neck involvement = 13 (33%)
Biological drug discontinuation	N = 10 (26%)
Treatment duration (median, months)	N = 8 4.5 (range, 1–18)
Outcome	Stability = 16 (41%) Repigmentation = 14 (36%) Progression = 3 (8%) n/r = 6 (15%)

n/r, not reported.

Thirty-nine patients with a mean age of 43 years (standard deviation ± 16.3) were identified, including 24 men (62%) and 15 women (38%). Overall, tumor necrosis factor-alpha (TNFα) inhibitors (infliximab, adalimumab, and certolizumab pegol) were the most frequently reported culprit drugs, accounting for more than half of the patients, followed by the anti-interleukin (IL)-17A antibodies ixekizumab and secukinumab in 13% and 8% of the patients, respectively. The anti-IL-12/23 ustekinumab and the anti-IL-4/13 dupilumab were each administered in four patients (10% of the total). Abatacept, a CTLA-4-Ig fusion protein that inhibits T-cell co-stimulation, was identified as the suspected biological drug in one patient (18). In many patients, no family history of vitiligo was reported. The median time to vitiligo onset was 5 months, ranging from 1.5 to 24 months after biological therapy initiation (**Table 2**); face and neck involvement was described in one-third of the cases (33%), and, interestingly, two patients presented with segmental vitiligo in the right mandibular area and right upper trunk (14, 17). In 10 patients (26% of the cases), vitiligo onset led to biological drug discontinuation or switching, while different treatment strategies were implemented, including topical steroids, topical calcineurin inhibitors, cyclosporine, and excimer laser (**Table 1**). Treatment duration was available for only 8 of 39 patients (20.5%), yielding a median duration of 4.5 months (range, 1.0–18.0). Regarding therapeutic outcomes, 41% of the patients had disease stabilization, 36% experienced varying degrees of repigmentation, and 8% of the cases progressed; follow-up information was not reported in the remaining 15% of the patients.

## Discussion

Based on the literature search, the occurrence of *de novo* vitiligo following biological therapy appears anecdotal, with most evidence consisting of a single case report and only two case series on the topic. No relevant data on risk factors, including sex, age, specific drug class, and clinical characteristics associated with vitiligo onset, could be inferred from these data. The comorbid dis-immune disease for which the biological drug was administered represents the common risk factor across cases (**Table 1**). In the next sections, we provide a critical review of the association between vitiligo and the specific class of drugs and comorbid disease.

### *De novo* vitiligo and TNFα inhibitors

As stated above, more than half of the *de novo* vitiligo cases were associated with TNFα inhibitors: 33% of the cases received adalimumab (13 patients), 16% received infliximab (6 patients), and 8% received certolizumab pegol (3 patients) (**Table 2**). Although this class of drug is known to trigger paradoxical immune reactions, most frequently psoriasis, vitiligo is not listed as a cutaneous or immune-related side effect in the summary of product characteristics, even in post-marketing analyses (30–32). The mechanism underlying immune-related cutaneous paradoxical reactions induced by TNFα inhibitors has been most extensively studied in psoriasis, where it is believed to block the maturation of dermal plasmacytoid dendritic cells (pDCs) (33). This inhibition results in an unbalanced production of type I interferons, potentially contributing to disease onset (33, 34).

Indeed, both type I interferons during the initial phase of the disease and IFN-γ, together with TNFα during the late-stage disease, play a crucial role in vitiligo pathogenesis. This role is also supported by the recent approval of topical ruxolitinib cream, which reduces the IFN-γ downstream pathway via Janus kinase 1/2 (JAK)/STAT inhibition (1). By contrast, it has been demonstrated that elevated local and systemic TNFα levels have been associated with vitiligo clinical activity (35–37), although direct inhibition of TNFα yielded unsatisfactory and heterogeneous results in vitiligo repigmentation based on pilot trials (38). Interestingly, a 10-year Korean population study found a nearly twofold increase in vitiligo risk among patients treated with TNFα inhibitors, with age <40 representing a major risk factor in the subgroup analysis (39). Based on these real-life observations, although molecular and experimental evidence regarding vitiligo is currently lacking, it may be hypothesized that TNFα inhibitors might trigger vitiligo onset in predisposed individuals.

However, it should be noted that all the patients received TNFα inhibitors for a concomitant autoimmune disease (**Table 1**). Indeed, a current bidirectional intrinsic risk exists between Inflammatory Bowel Diseases (IBDs) and vitiligo, as Crohn’s disease (CD) and ulcerative colitis (UC) patients are at major risk of developing vitiligo compared to the general population due to the common autoimmune predisposition (40, 41). Similarly, based on the common immune background, vitiligo is also associated with inflammatory uveitis and rheumatoid arthritis (1, 42, 43), thus implying that being affected by an autoimmune disease carries an independent, higher risk of developing vitiligo.

### *De novo* vitiligo and anti-IL-17 and IL-12/23 agents

*De novo* vitiligo was reported in 13% (n = 5) and 8% (n = 3) of the patients receiving the anti-IL-17A ixekizumab and secukinumab, respectively, and in 10% (n = 4) receiving the anti-IL-12/23 ustekinumab (**Table 2**). As for anti-TNFα medications, vitiligo is not listed as a cutaneous adverse event in the summary of product characteristics of anti-IL-17 and anti-IL-12/23 agents (44–46). The role of T helper (Th) 17 cells in vitiligo pathogenesis has been questioned by a pilot trial investigating secukinumab efficacy in active vitiligo patients, which had to be prematurely stopped due to disease progression in the majority of the patients (47). The authors demonstrated that a predominant subset of Th1-skewed cells was associated with active vitiligo instead of IL-17-secreting Th17 lymphocytes (47). This trial currently represents the only theoretical model that could link IL-17 inhibition to vitiligo onset or progression due to a skewed Th1 response, although a mechanistic causality could not be established based on the existing data. Similarly, a 24-week pilot trial of the anti-IL-23 tildrakizumab for stable vitiligo did not show statistically significant repigmentation, falling short of its primary endpoints (48).

Of note, in all patients identified in our literature search, anti-IL-17 and anti-IL-12/23 agents were prescribed to treat concurrent psoriasis, which is associated with a threefold risk of developing vitiligo based on a recent meta-analysis (49), and conversely, the prevalence of psoriasis in vitiligo patients is estimated to be between 2% and 5% (1).

### *De novo* vitiligo and anti-IL-4/IL-13 dupilumab

Overall, 10% (four individuals) of the patients in our literature search developed *de novo* vitiligo after exposure to the anti-IL-4/13 dupilumab, which had been prescribed for moderate-to-severe atopic dermatitis (**Table 2**). Regarding this drug class, no major immune-related adverse events, including vitiligo, have been described in the literature or in the summary of product characteristics (50). Moreover, experimental studies correlating IL-4 and/or IL-13 inhibition to vitiligo development are lacking; a possible Th1 immunological skewing favored by Th2 inhibition, similar to IL-17 inhibition, can only be hypothesized as a proposed pathomechanism (28). Stronger evidence supports the association between atopic disorders and vitiligo (1): a questionnaire involving 2,848 vitiligo patients showed that 61.8% reported at least one atopic comorbidity, with 24% suffering from atopic dermatitis (AD) (51), while a meta-analysis confirmed an increased risk of vitiligo in both adults and pediatric AD patients (odds ratio of 4.46 and 2.83, respectively) (52).

## Strength and limitations

This scoping review has several limitations. First, the available evidence mainly relies on single case reports and small case series, thereby precluding definitive conclusions on causality, incidence, risk factors, or class-specific effects. Second, reporting was heterogeneous across the included articles, with incomplete information especially on treatment duration, follow-up length, concomitant therapies, and objective assessment of disease activity or repigmentation. Finally, the presence of underlying immune-mediated diseases in most patients represents an important confounding factor, making it difficult to distinguish a drug-induced event from the intrinsic risk of vitiligo associated with comorbid dis-immune disorders.

Despite these inherent limitations, our scoping review is, to date, among the first to critically analyze drug-induced vitiligo through a structured and analytical approach to provide a deeper mechanistic interpretation of this phenomenon. Ultimately, this work is intended to serve as a practical guide to orient researchers within the current literature and to stimulate more targeted and high-quality research to fully elucidate this debated topic.

## Conclusion

Based on our results, although *de novo* vitiligo has been described as a possible drug-related adverse event during biological therapies targeting inflammatory cytokines, no strong experimental or clinical evidence exists to establish a causal relationship. While the suspected association with TNFα inhibitors may be plausible due to the established immune-related paradoxical reactions of these drugs and a single population-based study (40), in cases involving other monoclonal antibodies (ixekizumab, secukinumab, ustekinumab, and dupilumab), the association appears anecdotal.

Notably, the intrinsic risk of developing vitiligo in individuals affected by a co-occurring dis-immune comorbidity for which the biological agent is administered represents the only common risk factor among the cases; this suggests that drug association in these cases may be coincidental.

It could be hypothesized that biological drugs may act as a vitiligo trigger in predisposed individuals, but further studies involving large patient cohorts and experimental investigations are needed to fully elucidate this phenomenon. Such research could enable patient risk stratification, facilitating prompt diagnosis and optimized therapeutic management of the possible adverse event.

## Data Availability

The original contributions presented in the study are included in the article/supplementary material. Further inquiries can be directed to the corresponding author.
